# Higher Blood Lead Level Is Associated With Increased Likelihood of Abdominal Aortic Calcification

**DOI:** 10.3389/fcvm.2021.747498

**Published:** 2021-10-14

**Authors:** Zheng Qin, Hancong Li, Yingfei Xu, Jiameng Li, Baihai Su, Ruoxi Liao

**Affiliations:** ^1^Department of Nephrology, National Clinical Research Center for Geriatrics, West China Hospital, Sichuan University, Chengdu, China; ^2^Med+ Biomaterial Institute of West China Hospital, West China School of Medicine of Sichuan University, Chengdu, China; ^3^Med-X Center for Materials, Sichuan University, Chengdu, China; ^4^West China School of Medicine, West China Hospital of Sichuan University, Chengdu, China; ^5^Hefei Institutes of Physical Science, Chinese Academy of Sciences, Hefei, China; ^6^University of Science and Technology of China, Hefei, China

**Keywords:** lead poisoning, blood lead level, abdominal aortic calcification, NHANES, cross-sectional study

## Abstract

**Aims:** This study aimed to evaluate the association between blood lead level (BLL) and abdominal aortic calcification (AAC) in US adults aged ≥40 years.

**Methods:** We obtained data from 2013 to 2014 National Health and Nutrition Examination Survey (NHANES). Participants missing the data of BLL and AAC scores were excluded. BLL was measured using inductively coupled plasma mass spectrometry directly. AAC scores were quantified by Kauppila score system, and severe AAC was defined as AAC score >6. Weighted multivariable regression analysis and subgroup analysis were conducted to explore the independent relationship between BLL with AAC score and severe AAC.

**Results:** A total of 1,530 participants were included with the mean BLL of 1.45 ± 1.31 ng/dl and mean AAC score of 1.40 ± 3.13. The prevalence of severe AAC was 7.98% overall, and participants in higher BLL quartile showed higher prevalence of severe AAC (Quartile 1: 3.55%, Quartile 2: 7.28%, Quartile 3: 9.88%, Quartile 4: 12.58%, *P* < 0.0001). BLL was positively associated with higher AAC score (β = 0.15, 95% CI: 0.02, 0.27, *P* = 0.021) and increased risk of severe AAC (OR = 1.11; 95% CI: 1.00–1.22; *P* = 0.047). Subgroup analysis and interaction test indicated that the association between BLL and AAC was similar in different population settings.

**Conclusions:** Higher BLL was associated with higher AAC score and increased risk of severe AAC. Lead burden should be considered for people with AAC in clinical settings.

## Introduction

Vascular calcification (VC) is the abnormal deposition of calcium, phosphorus, and other minerals in the vessel wall and could be commonly observed in patients with chronic kidney disease (CKD) and diabetes (1–3). It is also one of the most significant independent risk factor for cardiovascular disease (CVD) and mortality, especially in CKD population (4, 5). The development of VC is partly related to the disorder of mineral metabolism such as hyperphosphatemia, which induces phenotypic transformation of vascular smooth muscle cells (VSMCs) into collagen-secreting osteoblasts (6). Kidney Disease: Improving Global Outcomes (KDIGO) recommended that patients with CKD stage 3–5 who have vascular or valve calcification should be considered to have the highest risk of CVD, which has been reported 20–30 times higher increased risk than health population (7, 8). In the past decades, the primary focus of VC was mostly coronary artery calcification, but more recently, there has been increased attention on abdominal aortic calcification (AAC) (9). The prevalence of AAC increased with age, ranging from 60% at aged 65–69 years to 96% at 85 years and older (10). AAC is a marker of subclinical atherosclerosis and has been recognized as a good predictor of cardiovascular events. Previous studies have reported that AAC deposition detected on lateral lumbar radiographs was an independent predictor of subsequent cardiovascular morbidity and mortality (4). In another meta-analysis including 10 studies, AAC was a strong predictor of CVD and mortality in the general population, especially in those with higher aortic calcification levels (11).

In order to evaluate the severity of calcified vessels, Kauppila et al. (12) developed an AAC grading quantification method (AAC scores) by using lateral lumbar radiography and found it could predict all-cause death and CVD in hemodialysis patients independently. A higher AAC score corresponds to a more serious calcified vascular condition. In 2009, KDIGO recommended that AAC score could reflect aortic calcification condition and the management based on AAC score was necessary for peritoneal dialysis patients (13).

Lead exposure is one of the most common toxic environmental diseases. In United States, more than 3 million workers were reported to at risk of occupational lead exposure (14). Lead exposure routes also include some food and water sources such as seafood, rice, and so on (15–19). The half-life of lead in the blood was thought to be 27–36 days, and the lead exposure could be daily, thus leading to a chronic damage and health burden (20). Lead exposure could damage the heart and blood vessels, thus increasing the risk of hypertension and cardiovascular disease by increasing oxidative stress, altering the function of blood vessel cells, inducing inflammatory responses, and disrupting calcium homeostasis (21–23).

Previous studies have reported that elevated blood lead level (BLL) could increase the risk of CVD. A study evaluating the combined effect of lead exposure and allostatic load on cardiovascular mortality demonstrated that higher BLL positively associated with elevated blood pressure and increased the likelihood of cardiovascular disease mortality with the interaction of allostatic load (24). Another study in a cohort of community-based older men suggested a positive association between blood and bone lead concentration with hypertension (25). A study based on data from NHANES 1999–2016 also reported that BLL was associated with higher prevalence of hypertension and uncontrolled hypertension, especially in men (26). However, the relationship between blood lead and AAC has not been reported before.

Therefore, using data from the National Health and Nutrition Examination Survey (NHANES), the aim of this study was to explore the relationship between blood lead and AAC, which may shed new light on the management and intervention of AAC in clinical practice. We hypothesized that BLL was positively associated with AAC score and the risk of severe AAC.

## Materials and Methods

### Study Population

We obtained data from NHANES, a cross-sectional study aimed to assess the health and nutrition status of the U.S. population conducted by the National Center for Health Statistics (NCHS) of the U.S. Center for Disease Control and Prevention (CDC). NHANES is still going on with a 2-year repeated cycle, and the survey data is updating as well. Due to the stratified multi-stage probability method adopted in the NHANES study design, its enrolled participants showed a relatively great representativeness (27). All NHANES data are publicly available at https://www.cdc.gov/nchs/nhanes/.

**Figure 1:**
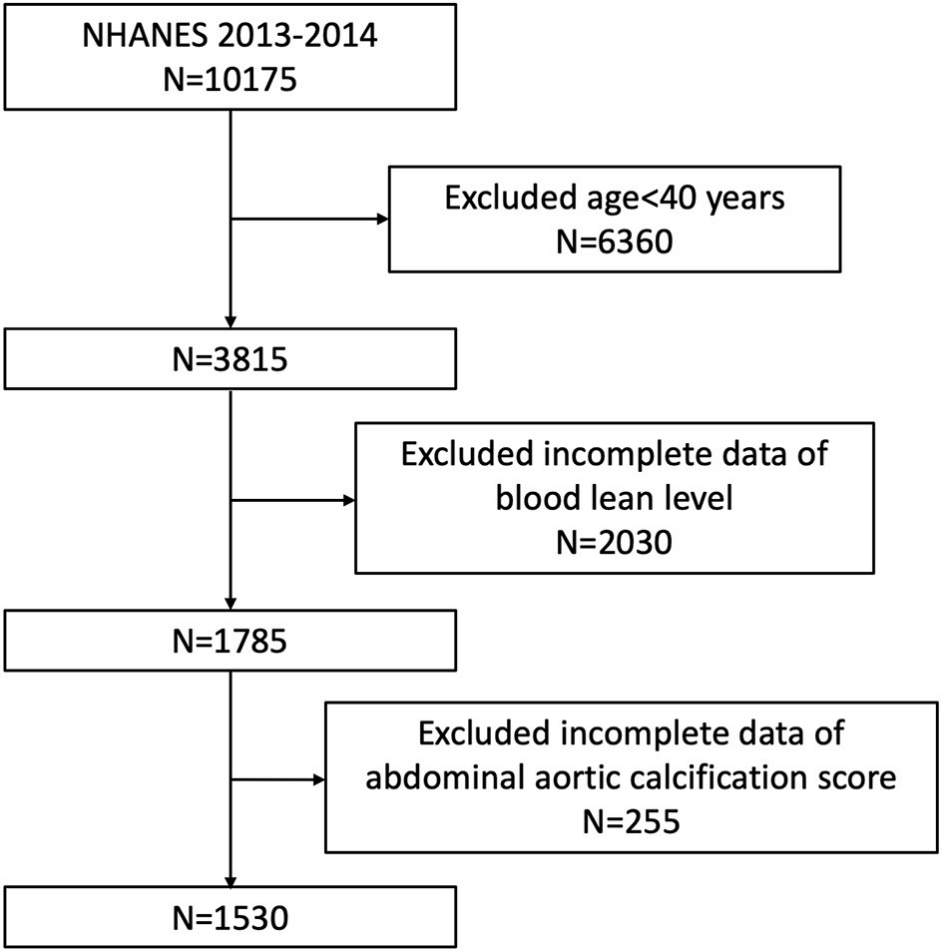
Flowchart of the sample selection from NHANES 2013–2014.

Our study was based on NHANES 2013–2014, since only this survey cycle included data both on blood lead and AAC score. Because data about AAC score was not available for participants aged <40 years old (they were excluded for DXA scan in NHANES 2013–2014), we enrolled participants aged ≥40 years with complete data about blood lead and AAC score in our analysis. A total of 10,175 individuals were enrolled at first, after exclusion of participants aged <40 years old (*n* = 6,360), missing the complete data about BLL (*n* = 2,030) and AAC score (*n* = 255), 1,530 subjects aged ≥40 years were included in our final analysis (Figure 1).

The NCHS Ethic Review Board granted the human subject approval for the conduction of NHANES, and written informed consent was obtained from all participants.

### Exposure and Outcome Definitions

BLL was designed as exposure variable. Venous whole blood samples were collected by phlebotomists in and analyzed in the Division of Laboratory Sciences, National Center for Environmental Health for CDC. The concentration of lead of whole blood specimens was measured using inductively coupled plasma mass spectrometry directly after a simple dilution sample preparation step as previous described (28). It was noted that the blood lead was above the detection limit for all non-missing values in our analysis. The variable code used for blood lead concentration was LBXBPB in NHANES.

AAC score and severe AAC were designed as outcome variables. AAC score was used to evaluate the severity of calcified abdominal aorta. A higher AAC score corresponded to a more severe calcification condition. It was quantified according to the Kauppila score system by assessing lateral lumbar spine images obtained using dual-energy X-ray absorptiometry (DXA, Densitometer Discovery A, Hologic, Marlborough, MA, USA) (12). In the Kauppila scoring method for AAC, different calcification severity of each segments of aortic walls corresponding to the region in front of the lumbar vertebrate L1-L4 was quantified to different scores according to the calcific deposit proportions and the sum of scores for these four segments was the total AAC scores, which ranged from 0 to 6 for each segment and 0 to 24 for the total AAC score (12). Total AAC score was designed as an outcome variable in our study and was obtained directly from NHANES data, which scored after reading the participant's DXA scans images by an NHANES affiliated staff member. The variable code used for total AAC score was DXXAAC24 in NHANES. In addition, we defined severe AAC as a total AAC score > 6, which was considered as a significant calcified abdominal aorta according to previous studies (1, 12, 29).

### Covariates

Potential covariates may confound the association between BLL and AAC were summarized in our multivariable-adjusted models based on previous studies (26, 30–32). Continuous variables in our study included age, systolic blood pressure (SBP), diastolic blood pressure (DBP), serum creatinine, serum cotinine, hemoglobin A1c, serum uric acid, serum calcium, serum phosphorus, total cholesterol, and total 25-hydroxyvitamin D. Categorical variables included gender, race, education level, body mass index (BMI), hypertension, and diabetes. BMI was categorized as <25, 25–29.9, and ≥30 kg/m^2^, which corresponded to normal weight, overweight, and obese population. All detailed measurement processes of these variables were publicly available at https://www.cdc.gov/nchs/nhanes/.

### Statistical Analysis

All statistical analysis was conducted according to CDC guidelines, and an appropriate NHANES sampling weights was applied which accounted for complex multistage cluster survey design in the analysis (33).

Continuous variables were presented as mean with standard deviation and categorical variables were presented as percentage. Either a weighted student's *t* test (for continuous variables) or weighted chi-square test (for categorical variables) was used to evaluate the differences in groups divided by different BLL quartiles. Multivariate logistic regression models were employed to explore the independent relationship between BLL and AAC (including AAC score and severe AAC) in three different models. In model 1, no covariates were adjusted. Model 2 was adjusted for gender, age, and race. Model 3 was adjusted for gender, age, race, education level, body mass index, systolic blood pressure, diastolic blood pressure, serum creatinine, serum cotinine, hemoglobin A1c, serum uric acid, serum calcium, serum phosphorus, total cholesterol, total 25-hydroxyvitamin D, hypertension, and diabetes status. Subgroup analysis stratified by race, gender, age, BMI, hypertension, and diabetes was also conducted by stratified multivariate regression analysis. Additionally, an interaction term was added to test the heterogeneity of associations between the subgroups. *P* < 0.05 was considered statistically significant. All analysis was performed using Empower software (www.empowerstats.com; X&Y solutions, Inc., Boston MA) and R version 3.4.3 (http://www.R-project.org, The R Foundation).

## Results

### Baseline Characteristics of Participants

The weighted demographic baseline characteristics of included participants were shown in Table 1. A total of 1,530 participants were included, of whom 48.71% were male and 51.29% were female with the average age of 57.24 ± 11.54 years old. The mean of BLL was 1.45 ± 1.31 ng/dl overall, and the ranges of BLL quartiles 1–4 were 0.16–0.80, 0.81–1.21, 1.22–1.84, and 1.85–24.60 ng/dl, respectively. Among different quartiles of BLL, significant differences were observed in age, gender, BMI, SBP, DBP, diabetes, hypertension, serum creatinine, serum cotinine, serum uric acid, serum calcium, serum phosphorus and total cholesterol, AAC scores, and the prevalence of severe AAC. Both the AAC scores and the prevalence of severe AAC increase with the higher BLL quartiles. The mean AAC score was 1.40 ± 3.13 overall, and 0.70 ± 2.25, 1.44 ± 3.18, 1.60 ± 3.36, and 2.01 ± 3.59 for Quartiles 1, 2, 3, and 4. The prevalence of severe AAC was 7.98% for the whole participants, and participants in higher BLL quartile trended to show higher rates of severe AAC (Quartiles 1: 3.55, 2: 7.28, 3: 9.88, 4: 12.58%, *P* < 0.0001).

**Table 1:** Baseline characteristics of participants, weighted.

**BLL**	**Overall**	**Quartile 1(0.16–0.80)**	**Quartile 2 (0.81–1.21)**	**Quartile 3 (1.22–1.84)**	**Quartile 4 (1.85–24.60)**	***P*-value**
Age (year)	57.24 ± 11.54	52.04 ± 10.27	57.45 ± 11.18	59.76 ± 11.55	61.00 ± 11.13	<0.0001
**Gender (%)**						
Male	48.71	35.56	52.81	50.44	58.36	<0.0001
Female	51.29	64.44	47.19	49.56	41.64	
**Race (%)**						
Mexican American	6.99	8.40	6.17	7.39	5.87	0.067
Other Hispanic	4.45	6.55	2.75	4.73	3.67	
Non-Hispanic White	71.77	70.55	75.49	67.77	72.56	
Non-Hispanic Black	9.82	9.34	8.25	10.48	11.76	
Other Races	6.97	5.16	7.34	9.63	6.14	
**Education level (%)**						
Less than high school	5.60	4.60	3.72	7.11	7.72	0.104
High school or GED	31.02	28.55	31.74	32.16	32.08	
Above high school	63.38	66.85	64.53	60.74	60.20	
**BMI (%)**						
Normal weight	26.54	22.66	20.10	29.00	34.28	<0.0001
Overweight	39.02	30.79	44.98	39.34	40.00	
Obese	34.43	46.55	34.93	31.66	25.72	
SBP (mmHg)	125.02 ± 17.37	122.02 ± 14.72	124.52 ± 17.10	126.26 ± 17.28	128.13 ± 20.01	<0.0001
DBP (mmHg)	70.81 ± 13.01	72.58 ± 11.76	71.19 ± 11.16	69.43 ± 14.14	69.50 ± 15.02	0.0027
Diabetes (%)	12.52	14.39	15.29	9.18	9.96	0.020
Hypertension (%)	43.47	37.40	44.37	44.07	49.36	0.010
Serum creatinine (mg/dl)	0.93 ± 0.47	0.86 ± 0.20	0.92 ± 0.25	0.90 ± 0.21	1.08 ± 0.91	<0.0001
Serum cotinine (ng/ml)	57.71 ± 141.83	31.77 ± 97.33	51.45 ± 141.65	82.70 ± 177.15	73.87 ± 143.59	<0.0001
Hemoglobin A1c (%)	5.74 ± 0.97	5.70 ± 1.00	5.77 ± 0.96	5.81 ± 1.12	5.68 ± 0.78	0.24
Serum uric acid (μmol/L)	319.09 ± 81.61	298.26 ± 77.65	326.48 ± 86.97	317.23 ± 79.76	338.37 ± 74.78	<0.0001
Serum calcium (mmol/L)	2.37 ± 0.09	2.35 ± 0.08	2.37 ± 0.09	2.37 ± 0.08	2.38 ± 0.11	<0.0001
Serum phosphorus (mmol/L)	1.23 ± 0.19	1.21 ± 0.16	1.22 ± 0.18	1.26 ± 0.20	1.23 ± 0.20	0.0004
Total cholesterol (mmol/L)	5.10 ± 1.06	4.97 ± 0.98	5.12 ± 1.12	5.16 ± 0.97	5.17 ± 1.14	0.035
Total 25-hydroxyvitamin D (nmol/L)	75.99 ± 29.09	75.29 ± 29.82	77.08 ± 28.27	76.83 ± 32.16	74.63 ± 25.65	0.60
Blood lead level (μg/dl)	1.45 ± 1.31	0.60 ± 0.14	0.99 ± 0.11	1.45 ± 0.17	3.08 ± 1.97	<0.0001
AAC score	1.40 ± 3.13	0.70 ± 2.25	1.44 ± 3.18	1.60 ± 3.36	2.01 ± 3.59	<0.0001
Severe AAC (%)	7.98	3.55	7.28	9.88	12.58	<0.0001

*GED, general educational development; RIP, ratio of family income to poverty; BMI, body mass index; SBP, systolic blood pressure; DBP, diastolic blood pressure; AAC, abdominal aortic calcification*.

### Higher BLL Is Associated With Higher AAC Score and Increased Risk of Severe AAC

A positive association between BLL and AAC scores was observed (Model 1: β = 0.22, 95% confidence interval, CI: 0.10–0.34, *P* = 0.0002; Model 2: β = 0.12, 95% CI: 0.00–0.24, P = 0.045). In the fully adjusted model (Model 3), this positive association was still stable (β = 0.15, 95% CI: 0.02, 0.27, *P* = 0.021), indicating that each unit of increased BLL was associated with 0.15 higher unit of AAC score. We also converted BLL from a continuous variable to a categorical variable (quartiles) to conduct sensitivity analysis. AAC score increased with the higher BLL quartiles group. The average AAC score of Quartiles 2, 3, and 4 was 0.58, 0.60, and 0.99 unit higher compared with the lowest quartile (Quartile 1), respectively (Table 2).

**Table 2:** Association of blood lead level with AAC score and severe AAC.

**Blood lead level**	**β^**1**^**/OR**^**2**^**(95% CI**^**3**^**)**, ***P*****-value****		
	**Model 1^**4**^**	**Model 2^**5**^**	**Model 3^**6**^**
**AAC score**			
Continuous	0.22 (0.10, 0.34) 0.0002	0.12 (0.00, 0.24) 0.045	0.15 (0.02, 0.27) 0.021
**Categories**			
Quartile 1	Reference	Reference	Reference
Quartile 2	0.74 (0.33, 1.16) 0.0005	0.32 (-0.09, 0.73) 0.12	0.58 (0.15, 1.02) 0.0089
Quartile 3	0.90 (0.46, 1.34) <0.0001	0.34 (-0.09, 0.78) 0.12	0.60 (0.14, 1.07) 0.011
Quartile 4	1.31 (0.87, 1.75) <0.0001	0.79 (0.35, 1.22) 0.0004	0.99 (0.50, 1.48) <0.0001
**Severe AAC**			
Continuous	1.10 (1.00, 1.20) 0.043	1.11 (1.01, 1.22) 0.024	1.11 (1.00, 1.22) 0.047
**Categories**			
Quartile 1	Reference	Reference	Reference
Quartile 2	2.29 (1.23, 4.28) 0.0093	2.14 (1.14, 4.02) 0.018	1.68 (0.86, 3.25) 0.13
Quartile 3	2.42 (1.30, 4.51) 0.0054	2.49 (1.33, 4.68) 0.0046	2.15 (1.10, 4.19) 0.025
Quartile 4	3.85 (2.13, 6.97) <0.0001	3.90 (2.13, 7.13) <0.0001	3.72 (1.94, 7.12) <0.0001

*In sensitivity analysis, blood lead level was converted from a continuous variable to a categorical variable (quartiles). ^1^β: effect sizes; ^2^OR: odd ratio; ^3^95% CI: 95% confidence interval; ^4^Model 1: no covariates were adjusted; ^5^Model 2: adjusted for gender, age and race; ^6^Model 3: adjusted for gender, age, race, education level, body mass index, systolic blood pressure, diastolic blood pressure, serum creatinine, serum cotinine, hemoglobin A1c, serum uric acid, serum calcium, serum phosphorus, total cholesterol, total 25-hydroxyvitamin D, hypertension, and diabetes. AAC, abdominal aortic calcification*.

As for the occurrence of severe AAC, we found that increased BLL was associated with a higher risk of severe AAC (Model 1: OR = 1.10; 95% CI:1.00–1.20; *P* = 0.043; Model 2: OR = 1.11; 95% CI: 1.01–1.22; *P* = 0.024; Model 3: OR = 1.11; 95% CI: 1.00–1.22; *P* = 0.047). In Model 3 which adjusted for all covariates, our results indicated that each unit of increased BLL was associated with 11% increased risk of severe AAC. In sensitivity analysis, the adjusted OR (reference to Quartile 1) was 2.15 (95% CI: 1.10–4.19; *P* = 0.025) for Quartile 3 and 3.72 (95% CI: 1.94–7.12; *P* = < 0.0001) for Quartile 4, suggesting a stable positive relationship between higher BLL and increased risk of severe AAC with statistical significance (Table 2).

### Subgroup Analysis

Subgroup analysis was performed to evaluate the robustness of association between BLL and AAC. We also tested the interactions with race, gender, age, BMI, hypertension, and diabetes to evaluate that if there was any significant dependence of the effect modifier on this relationship. However, no correlation with the P for interaction meet the statistical significance was detected, indicating that there was no dependence on these effect modifiers for this association (all P for interaction >0.05).

**Figure 2:**
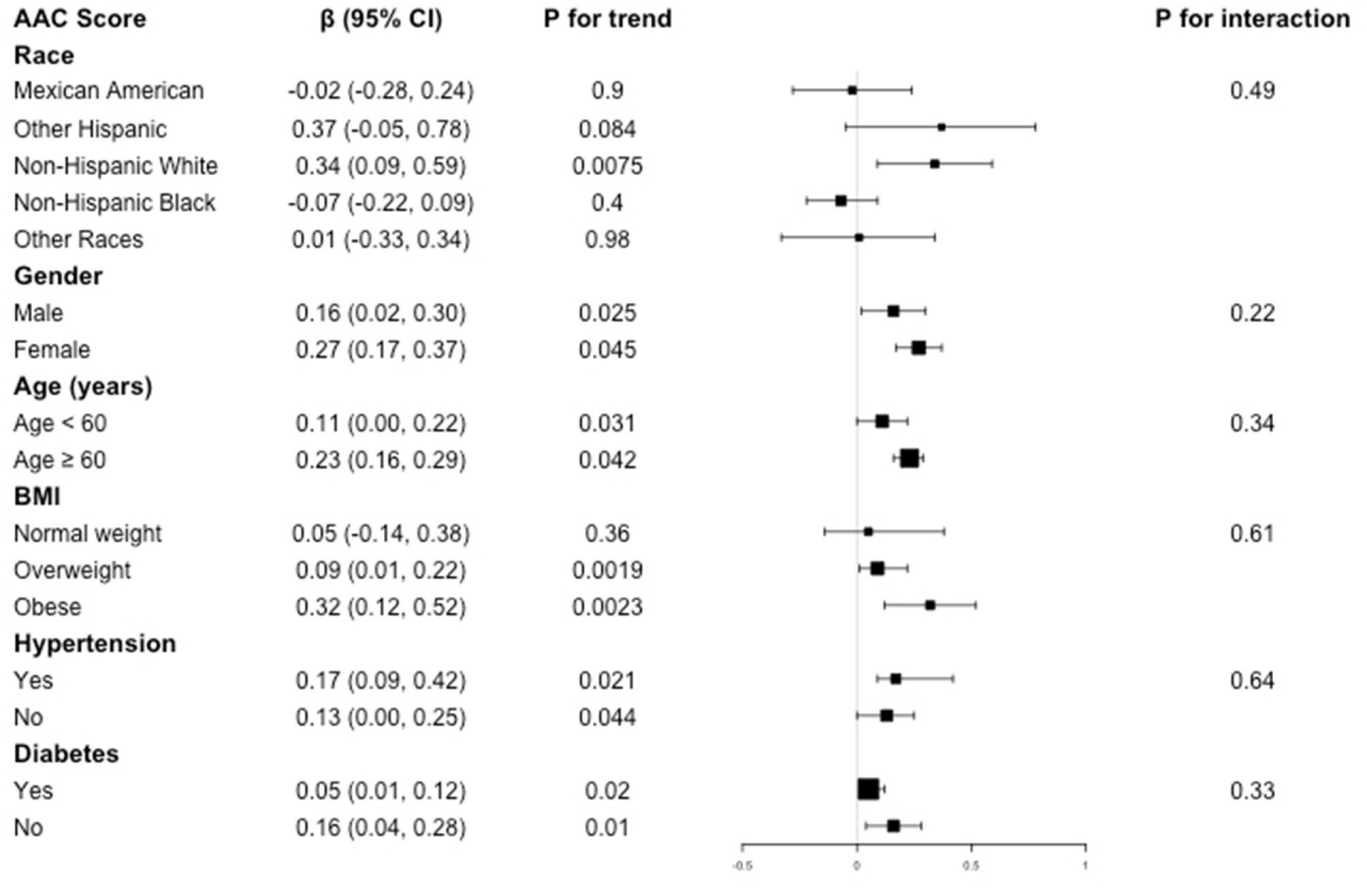
Subgroup analysis for the association between BLL and AAC score.

For the association between BLL and AAC score, in subgroup stratified by gender, age, hypertension, and diabetes, this positive association was stable (all P for trend < 0.05 and P for interaction >0.05). BLL positively associated with higher AAC score in overweight (β = 0.09, 95% CI: 0.01, 0.22, *P* = 0.0019) and obese population (β = 0.32, 95% CI: 0.12, 0.52, *P* = 0.0023), while this positive relationship did not meet the statistical significance in normal weight group (*P* = 0.36). However, interaction test results indicated that there was no dependence on BMI for this association (*P* for interaction = 0.61) (Figure 2).

**Figure 3:**
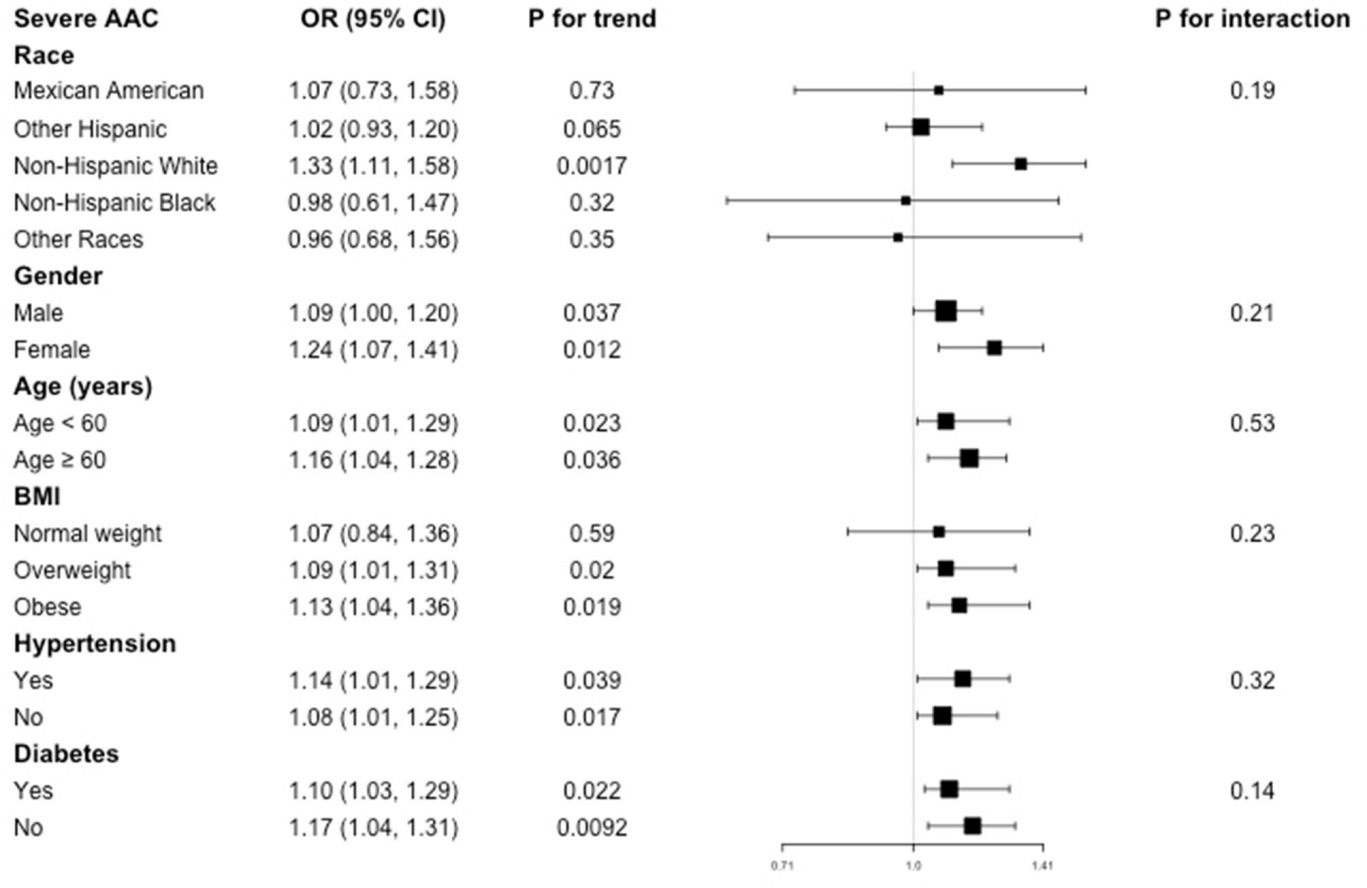
Subgroup analysis for the association between BLL and severe AAC.

As for the association between BLL and severe AAC, subgroup analysis showed the similar results. BLL was positively associated with increased risk of severe AAC in overweight and obese subgroup, but this positive association did not meet the statistical significance in normal weight participants (*P* = 0.59), with a P for interaction of 0.23. Similarly, we did not find any significant dependence on effect modifiers (all *P* for interaction >0.05). These results indicated that the positive correlation was similar in population with different race, gender, age, BMI, hypertension, and diabetes status and could be appropriate for various population setting as well (Figure 3).

## Discussion

In this cross-sectional study including 1,530 adults, we found that higher BLL was independently associated with higher AAC score and increased risk of severe AAC. For AAC score, compared with the lowest BLL quartile, higher BLL quartile trended to show higher AAC score (0.58, 0.60, and 0.99 unit higher for Quartile 2, 3, and 4) and higher risk of severe AAC (OR of 1.68, 2.15, and 3.72 for Quartile 2, 3, and 4), indicating that the association is driven not only by high blood lead. That is, increasing blood lead burden could augment the severity of aortic calcification condition. This association was similar in subgroups stratified by race, gender, age, BMI, hypertension, and diabetes status, suggesting that this association may be appropriate for different population setting. Our finding suggests that lead burden should be considered for people with AAC in clinical settings.

To our knowledge, this is the first study assessing the association between BLL and AAC. Previous studies have reported the association between BLL and CVD with varying epidemiological methods and target populations (18, 24, 34, 35). A population-based cohort with 18,602 participants aged 40 and above in America found a positive association between hematocrit- or hemoglobin-corrected blood lead and CVD mortality. To be specific, relative odds for CVD mortality associated with 10-fold increases for hematocrit- and hemoglobin-corrected blood were estimated to be 1.44 and 1.46, respectively (36). Similarly, a significant positive relationship between serum lead level and an increased risk of 10-year cardiovascular events has been reported. A doubling of serum lead was associated with the a 10% increased risk of 10-year CVD according to a population-based study with 9602 Korean participants included (37). Another cross-sectional study demonstrated that BLL was associated with higher prevalence of hypertension and uncontrolled hypertension, with more pronounced in men (26). However, there also have been several studies shown the opposite results (38, 39). A mendelian randomization study with 2,603 twins and their families in UK reported blood lead was unrelated to coronary artery disease, diabetes, and blood pressure (38). Another study with 15,431 subjects in NHANES III did not find consistent association between BLL and blood pressure across all demographic groups (39). In our study, consistent with most studies, higher BLL was independently associated with higher AAC score and increased risk of severe AAC, suggesting that blood lead load may have a significant negative impact on cardiovascular health.

The exact mechanism of the positive association between BLL and AAC still remains unclear. The damage of cardiovascular system caused by lead poisoning *via* increasing oxidative stress, enhancing inflammatory responses and disrupting calcium homeostasis maybe a possible explanation to support our results (21–23). Animal studies have shown that elevated lead could induce oxidative stress and get involved with hypertension in mice model (40, 41). In addition, lead can promote the production of reactive oxygen species (ROS) and oxidative stress by participating in Fenton- and Haber-Weiss-type reactions. By promoting oxidative stress, lead exposure lowers nitric oxide (NO) production and causes NO inactivation, thus downregulating soluble guanylate cyclase and reducing cGMP production. Then, the increasing cytosolic Ca2+ concentration in VSMCs could lead to heightening systemic vascular resistance, raising arterial pressure and promoting VC in turn (22). Oxidative stress could promote inflammation, fibrosis, and apoptosis by activating NF-kappa B pathway as well, which is the general transcription factor for numerous pro-inflammatory cytokines, chemokines, and adhesion molecules, thus participating in the calcification process. In addition, vascular activity and volume regulation hormones could also be affected by lead exposure, such as adrenergic system, endothelin, prostatin, and atrial natriuretic peptide, thus mediating vascular changes. Lead also regulates calcium signaling pathways through interaction with calcium ions and directly mediating the VSMCs differentiation to osteoblast-like cells, thus leading to calcified vessels (22).

Our results of subgroup analysis demonstrated that there was no dependence of race, gender, age, BMI, hypertension, and diabetes on this positive association between BLL with AAC scores and severe AAC (all *P* for interaction >0.05), indicating that these positive correlations were similar in different population settings. Consistent results have been reported for the analysis between lead exposure with CVD before. A meta-analysis included 58,518 subjects showed that there was no difference for the association between blood lead and blood pressure both in men and women (42). However, a study with 30,762 participants based on NHANES found that men maybe more vulnerable to the negative effect of lead burden than women (26). Specifically, the effect between BLL and uncontrolled hypertension in men was greater than that of women, not only due to the higher BLL in men in general, but also possibly owing to some potential gender based biological effects modification. Physiological differences, such as hormone environment of ovary and testis and sex chromosome, maybe the reasons of this difference (43). Health-seeking behavior might play a role as well, regarding a cross-sectional study in French adults found that women tended to have a better awareness of hypertension than men and their hypertension was better controlled (44).

This study has several strengths. Data in our study was based on NHANES, a nationwide population-based sampling survey with a standard protocol and all analysis was performed with consideration of an appropriate NHANES sampling weights, making the study more representative. Additionally, we adjusted confounding covariates and the selection of covariates was mainly based on previous studies to reduce the confounding bias. However, the limitations should also be noted. Firstly, due to cross-sectional study design, we could not obtain a causal relationship between BLL and AAC. Therefore, longitudinal studies with larger sample size maybe needed to clarify whether increased BLL precedes AAC. Secondly, although we adjusted for some potential covariates, we could not completely rule out the influence from other possible confounders. Finally, patients aged <40 years did not went through DXA in NHANES study design and their AAC scores were missing; thus, we could not analyze this association for a widely age group.

## Conclusion

Higher blood lead burden was associated with higher AAC score and increased risk of severe AAC. Considering the negative effect of lead exposure on the cardiovascular health, lead burden should be considered for people with AAC in clinical settings. However, further large-scale prospective studies are still needed to clarify the precise causality of this relationship.

## Data Availability Statement

The original contributions presented in the study are included in the article/supplementary material, further inquiries can be directed to the corresponding authors.

## Ethics Statement

The studies involving human participants were reviewed and approved by the NCHS Ethic Review Board. The patients/participants provided their written informed consent to participate in this study.

## Author Contributions

ZQ: data analysis and writing—original draft. HL: formal analysis and writing—original draft. YX: data analysis and software. JL: data analysis. BS: methodology, conceptualization, and funding acquisition. RL: conceptualization and writing—reviewing and editing. All the authors approved the final version.

## Funding

This work was supported by the National Natural Science Foundation of China [Grant No. 82000702], the Science and Technology Achievement Transformation Fund of West China Hospital of Sichuan University [Grant No. CGZH19006], the 1.3.5 project for disciplines of excellence from West China Hospital of Sichuan University [Grant No. ZYJC21010], National Clinical Research Center for Geriatrics, West China Hospital, Sichuan University [Grant No. Z2018B10], and Med+ Biomaterial Institute of West China Hospital/West China School of Medicine of Sichuan University [Grant No. ZYME20001].

## Conflict of Interest

The authors declare that the research was conducted in the absence of any commercial or financial relationships that could be construed as a potential conflict of interest.

## Publisher's Note

All claims expressed in this article are solely those of the authors and do not necessarily represent those of their affiliated organizations, or those of the publisher, the editors and the reviewers. Any product that may be evaluated in this article, or claim that may be made by its manufacturer, is not guaranteed or endorsed by the publisher.
